# Late introduction of fish and eggs is associated with increased risk of allergy development – results from the FARMFLORA birth cohort

**DOI:** 10.1080/16546628.2017.1393306

**Published:** 2017-11-07

**Authors:** K. Jonsson, M. Barman, H. K. Brekke, B. Hesselmar, S. Johansen, A.-S. Sandberg, A. E. Wold

**Affiliations:** ^a^ Food and Nutrition Science, Department of Biology and Biological Engineering, Chalmers University of Technology, Gothenburg, Sweden; ^b^ Department of Nutrition, Institute of Basic Medical Sciences, University of Oslo, Oslo, Norway; ^c^ Department of Paediatrics, Institute of Clinical Sciences, University of Gothenburg, Gothenburg, Sweden; ^d^ Paediatric Clinic, Skaraborg Hospital, Lidköping, Sweden; ^e^ Clinical Bacteriology Section, Department of Infectious Diseases, University of Gothenburg, Gothenburg, Sweden

**Keywords:** farmers, allergy, breastfeeding, complementary foods, introduction of solids, fish, eggs

## Abstract

The prevalence of allergy is markedly low in children growing up on farms. An increasing number of studies indicate that the timing of food introduction may affect allergy development. We aimed to investigate if protection against allergy in farm environments may be mediated through differences in food-introduction practices between farm and non-farm families, using an explorative approach. Twenty-eight farm and 37 non-farm children were included in the FARMFLORA birth cohort. Practices of breastfeeding and introduction of formulas and complementary foods were collected by questionnaires at 6, 12, and 18 months of age. Allergy was diagnosed by pediatricians at 3 years of age. The only difference in food-introduction practices observed between farm and non-farm children was an earlier introduction of nuts in farmers (median month: 11 [IQR: 8–6] in farmers, 15 [12–19] in non-farmers). One farm child (4%) and 10 non-farm children (27%) were allergic at 3 years of age. Lower risk of allergy development was associated with early exclusive breastfeeding (continuous variable; OR = 0.59, 95% CI: 0.39–0.89), but also having received eggs (OR = 0.08, 95% CI: 0.13–0.54) and fish (logistic regression not applicable, *P* = 0.01 in likelihood ratio testing [χ^2^]) at 10 months of age or earlier compared to later. Our results were not affected by reverse causation, as judged by a questionnaire sent to the families in retrospect. Timing of introduction of complementary foods is unlikely to contribute to the lower risk of allergy among farm children. Although early exclusive breastfeeding was associated with a lower rate of allergy development, postponed introduction of complementary foods might increase the risk of developing allergy. Owing to the limited sample size, our results are only indicative, but support prior findings.

## Background

Children growing up on small dairy farms have a remarkably low incidence of allergy compared with other children growing up in rural environments, but not on a farm [1–3]. Frequent contact with livestock and fodder [4] and consumption of unpasteurized milk [5] have been associated with protection. However, other factors may contribute, such as differences in diet between farming and non-farming families. We have previously shown in the FARMFLORA birth cohort that farming mothers consumed more full-fat dairy products and saturated fats and less margarines and vegetable oils during pregnancy and lactation than mothers living in the same rural area, but not on farms [6]. This dietary pattern was mirrored in the diet of the children at 1 year of age [7]. Both the mothers’ and the children’s consumption of margarines was weakly associated with development of allergy in the children at 3 years of age [6,7]. Consumption of margarine has previously been shown to be associated with increased risk of allergy development [8–11].

**Figure 1.**
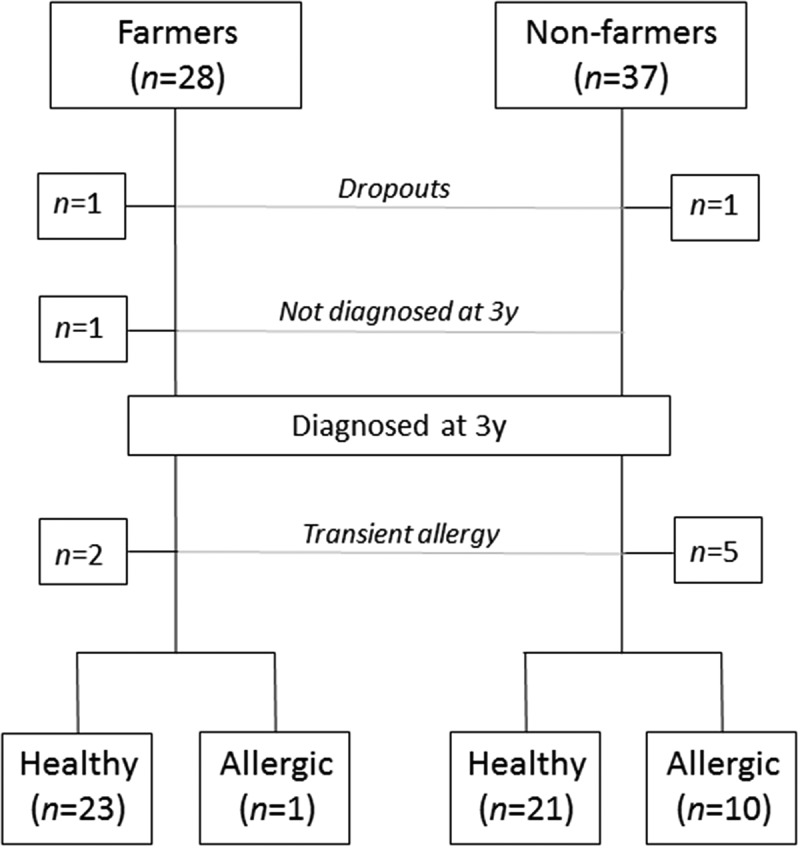
Flow chart of subjects eligible for analysis between healthy children and children allergic at 3 years of age. One farm and one non-farm family withdrew from the study due to personal reasons or change of residence. One farm child was not diagnosed at 3 years of age and two farm children and five non-farm children were diagnosed as allergic at 1.5 years of age but not at 3 years of age (transient allergy); hence, these children were neither included in the healthy nor the allergic group.

Another dietary factor that may affect allergy development is the timing of introduction of solid foods. Between the years 2000 and 2008, the American Academy of Pediatrics recommended parents in families with a history of allergy to postpone the introduction of dairy until the child was 1 year, eggs until 2 years, and fish, nuts, and peanuts until 3 years of age [12]. Introduction of complementary foods is currently recommended after the age of 4 to 6 months [13]. According to national recommendations, mothers in Sweden are advised to breastfeed exclusively for a period of 6 months [14]. However, such recommendations are in conflict with two recent randomized controlled studies [15,16]. One of the studies showed that introduction of peanuts between four and 11 months of age to high-risk infants dramatically reduced their risk of peanut allergy development [15]. The other study was conducted on the general population and showed that consumption of 2 g of protein from peanut or egg-white per week from the age of 3 months was associated with a lower prevalence of these respective allergies than was less consumption [16]. These findings agree with observations from studies in experimental animals; oral tolerance is induced to proteins fed in quite large amounts, while feeding miniscule amounts of foreign proteins may instead result in sensitization and allergy [17,18].

Apart from promoting oral tolerance and decreasing food allergy, early introduction of some complementary foods seems to protect against allergy in general, i.e. not only food allergy [19–21]. In particular, delayed introduction of fish has repeatedly been associated with a higher risk of developing different atopic diseases [22–29]. Accordingly, in the FARMFLORA birth cohort we found an inverse relationship between the proportions of the fish fatty acid eicosapentaenoic acid (EPA) in the sera of four-month-old infants and the risk of subsequent allergy development [30]. The proportions of EPA in the infants’ sera correlated with maternal breast milk EPA, which, in turn, correlated with the mother’s fish intake [30]. Also late introduction of eggs has been associated with an increased risk of developing asthma, allergic rhinitis, and atopic sensitization, as shown by Nwaru and coworkers [29]. Alongside these studies, a reduced risk of allergy development has been observed from introducing a broad variety of foods at an early age, i.e. a high food diversity [19,31–33].

The aim of the present study was to analyze whether differences in food-introduction practices may be part of the allergy protective effect observed in children growing up in a farming environment.

## Methods

### Subjects

Children from Skaraborg County in southwestern Sweden, 28 farm and 37 non-farm children from the same rural area but not living on farms, constituted the FARMFLORA birth cohort. Pregnant women were recruited between September 2005 and May 2008 at maternity clinics. Children born within gestational weeks 36–42 were included. In the farming group, at least one of the parents worked on a dairy farm, other types of farms were excluded. Non-farm families had to live in rural areas, but not on any type of farm.

Children underwent clinical examination for allergy diagnosis at 1.5 and 3 years of age. The study was based on the diagnosis made at 3 years of age, a decision taken by the study physicians prior to analysis. This decision was taken in order to obtain as clear diagnoses as possible, since rhinitis and asthma may be difficult to diagnose with certainty at a very young age. Children who obtained an allergy diagnosis at 1.5, but not at 3 years of age (transient allergy) were excluded, which included two farm and five non-farm children (Figure 1). In addition, one farm child that did not undergo clinical examination at 1.5 years was excluded. As a result, 55 children were included in the analysis of healthy and allergic subjects (Figure 1).

Written informed consent was provided from both parents. The study was approved by the Regional Ethics Committee in Gothenburg (No. 363-05).

**Figure 2.**
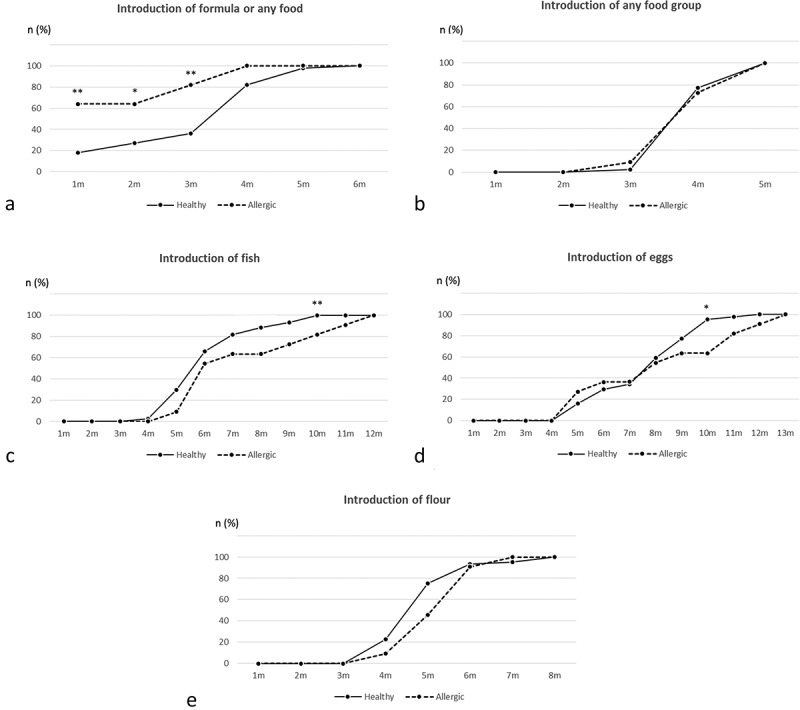
Cumulative rate of healthy and allergic children for whom formula or food have been introduced at different time-points. Numbers were calculated by χ^2^ tests. Analyses were carried out for introduction of formula or any food (a), any food (b), fish (c), eggs (d) and flour (e). Significant differences between healthy and subsequently allergic children are denoted as stars of significance: *P ≤ 0.05, **P ≤ 0.01.

### Dietary assessment

The parents recorded practices of breastfeeding, formulas and complementary foods continuously in diaries that were collected when the child was 6, 12, and 18 months old. Length of any and exclusive breastfeeding was registered in months. The month of introduction of any formula or complementary food was noted. The type of formula was recorded, including: milk based, gluten based, milk free, and gluten free, the latter two being considered hypoallergenic formulas. Each type of food was recorded separately and grouped as follows: potatoes, vegetables, fruits, berries, nuts, peanuts, legumes, eggs, fish, meat, dairy, and flour. The food diversity was calculated as the number of food groups that had been introduced at 6 months of age.

### Clinical examination

The children were examined clinically by pediatricians at the age of 3 years to diagnose food allergy, eczema, asthma, and allergic rhinoconjunctivitis. Sensitization against inhalant allergens (Phadiatop) and common foods (Fx5 Food Mix) was assessed by blood tests (Phadia, Uppsala, Sweden). Food allergy was defined as immediate (≤2 h after intake of food) or late-onset (>2 h) reactions that improved rapidly following allergen elimination from the diet. The diagnosis was supported by an open food challenge test and/or a positive Fx5 Food Mix test (Phadia); ImmunoCAP tests (Phadia) were used to identify the allergen. Atopic eczema was diagnosed according to the criteria of Williams [34] or Hanifin and Rajka [35]. Asthma was diagnosed as: ≥3 wheezing episodes combined with either (a) eczema, allergic rhinoconjunctivitis, or food allergy, or (b) a clinical response to leukotriene antagonists or inhaled glucocorticoids. At least one wheezing episode had to have occurred after 2 years of age for an asthma diagnosis at the age of 3 years. Allergic rhinoconjunctivitis was defined as symptoms from the eyes or the nose after exposure to pollen or animal dander, combined with demonstration of allergen-specific IgE to the corresponding inhalant allergen (ImmunoCAP, Phadia).

### Statistical methods

An explorative statistical approach was used, i.e. a large number of analyses were carried out to find meaningful cut-offs for the timing of introduction of different food items or food groups. The multiple testing increases the risk of type I errors; however, due to our limited sample size, hence the low power, we did not control for multiple testing to minimize the risk of type II errors.

#### Univariable analysis

Due to small sample sizes and non-normal distributions of the variables, nonparametric tests were used and data are given as medians and interquartile ranges. Variables were tested for significance using the Mann–Whitney *U* test (continuous variables) and χ^2^ test or Fisher’s exact test (categorical variables). Two-tailed *P*-values of ≤0.05 were considered significant. Dietary variables of introduction were analyzed both as continuous variables (month of introduction) and as categorical variables for introduction up to a specific month versus introduction after. The number of food items at 6 months were analyzed only as a continuous variable. IBM SPSS Statistics version 19 (IBM Corporation, New York, NY) was used for the statistical analyses.

#### Multivariable analysis

Binary logistic regression and χ^2^ likelihood ratio test were used to control for covariation and confounding (SPSS Statistics version 19, IBM Corporation). Variables with *P *≤ 0.20 in univariable analysis of healthy versus allergic children (Table 1) were included as potential covariates, including maternal heredity, paternal smoking during the last month of pregnancy, cats or dogs in the house at recruitment, gestational week, cesarean section, birth weight, male gender and farm/non-farm family. Dietary variables, which were analyzed as month of introduction in the univariable analysis, were converted to consumption before the age of 1.5 years in the multivariable analysis to display decreased risks of early introduction instead of increased risks of late introduction. The time-point of 1.5 years was chosen, since the last food diary was collected at that age.

**Table 1.** Characteristics of farm versus non-farm children and healthy versus subsequently allergic children.

Variables	Farmers (*n* = 28)	Non-farmers (*n* = 37)	*P*^e^	Healthy (*n* = 44)	Allergic (*n* = 11)	*P*^e^
Antenatal characteristics						
Heredity^a^						
Mothers	7 (25%)	11 (30%)	0.68	9 (21%)	5 (46%)	0.12
Fathers	1 (4%)	12 (32%)	0.01	7 (16%)	3 (27%)	0.40
Maternal age at delivery, year	33 (21–42)	32 (22–41)	0.46^f^	32 (21–42)	34 (22–41)	0.71^f^
Education, level^b^ (1 = lowest, 5 = highest)						
Mothers	2 (1–5)	4 (1–5)	0.20^f^	4 (1–5)	3 (1–5)	0.78^f^
Fathers	2 (1–5)	2 (1–5)	0.02^f^	2 (1–5)	2 (2–4)	0.51^f^
Smoking during last mo of pregnancy						
Mothers	0 (0%)	1 (3%)	1.00	1 (2%)	0 (0%)	1.00
Fathers	1 (4%)	4 (11%)	0.38	2 (5%)	2 (18%)	0.18
Cats or dogs in house at recruitment	21 (75%)	19 (51%)	0.054	31 (71%)	5 (46%)	0.16
Siblings	18 (64%)	17 (46%)	0.15	23 (52%)	6 (55%)	0.89
Birth characteristics						
Gestational week^c^	40 (37–42)	39 (36–42)	0.13^f^	39 (36–42)	39 (36–41)	0.14^f^
Cesarean section	3 (11%)	7 (19%)	0.50	5 (11%)	4 (36%)	0.07
Birth weight^d^, g	3495 (2780–4740)	3618 (2440–4830)	0.78^f^	3560 (2440–4740)	3235 (2730–4830)	0.14^f^
Infant characteristics						
Male gender	10 (36%)	23 (62%)	0.04	22 (50%)	9 (82%)	0.09
Maternal fish oil intake, 4 months postpartum	2 (11%)	0 (0%)	0.18	2 (7%)	0 (0%)	1.00
Vitamin A and D supplements at age 1 year	17 (61%)	22 (60%)	0.92	30 (68%)	6 (55%)	0.49
Allergic at 3 year of age	1 (4%)	10 (32%)	0.02			
Farm group				23 (52%)	1 (9%)	0.02

Data are presented as *n* (%) or medians (ranges). A lower total number of subjects are shown for the Healthy/Allergic group than the Farm/Non-farm group due to drop-outs and allergy selection (Figure 1).

^a^Doctor’s diagnosed asthma, rhinitis, or atopic eczema.

^b^1 = Elementary school, 2 = upper secondary school 2–3 years or equivalent, 3 = qualified graduate from upper secondary engineering course, 4 = university ≤1 year, 5 = university >1 year.

^c^ *n* = 27 farmers and 36 non-farmers, and *n *= 43 healthy and 10 allergic subjects, respctively.

^d^ *n* = 36 non-farmers, and *n *= 10 allergic subjects.

^e^χ^2^-test (or Fisher’s exact test).

^f^Mann-Whitney *U* test.

**Table 2.** Duration of breastfeeding and introduction of complementary foods in farm and non-farm children.

	Farmers (*n *= 28)		Non-farmers (*n *= 37)		
	Median	IQR	Median	IQR	
Variables	months		months		*P*
Exclusive breastfeeding	4	3–4	3	1–4	0.19
Any breastfeeding	8	5–11	8	4–12	0.74
Any food group	4	4–5	4	4–4	0.15
Potatoes	4	4–5	4	4–5	0.33
Vegetables	5	4–6	5	4–5	0.30
Legumes	7	6–8	6	5–8	0.59
Fruit	5	4–6	5	4–5	0.97
Berries	6	5–8	7	6–9	0.25
Peanuts	19	13–19	19	18–19	0.07
Nuts	11	8–16	15	12–19	0.02
Meat	6	5–6	6	5–6	0.71
Eggs	8	6–10	8	6–9	0.97
Fish	6	5–8	6	5–7	0.85
Flour	5	4–6	5	5–6	0.86
Milk	5	4–5	5	4–6	0.68
	Number of food items introduced				
Food diversity at age 6 months	8	6–9	8	6–9	0.88

#### Reverse causation

The allergic children tended more often to have an allergic mother, but not an allergic father. Allergic parents might be more restrictive in introducing complementary food into the diet of their children. To control for such potential reverse causation we analyzed whether food-introduction practices differed between children and mothers with or without a history of allergy. No significant differences were found; hence, parental heredity was not further accounted for. To further assess whether delayed introduction might be due to allergy or suspected allergy in the child (reverse causation), we sent out a question by mail to the parents asking: ‘Did you intentionally wait to introduce certain foods to the child (e.g. fish, eggs, flour/gluten) due to a family history of allergy or early signs of allergy in the child?’ This mail was sent to the parents when the child was 8 years of age, in average. The questionnaire was sent to 55 families (children who were healthy or allergic at age 3 after exclusion of those specified above). A response was obtained from 39 families (71%), and none of these reported that they had delayed the introduction of any foods for reasons of allergy or suspected allergy in the child.

## Results

Characteristics of farm and non-farm children as well as healthy and allergic control children are shown in Table 1. Children from farming families were more often girls, had less often an allergic father, and the fathers had lower educational level compared with the families of the control children (Table 1). No significant differences were observed between healthy and allergic children (Table 1).

### Food-introduction practices in children from farming and control families

Medians and interquartile ranges of duration of breastfeeding, month of introduction of different food groups, and food diversity at 6 months of age are presented for farm and non-farm children in Table 2. The only difference found between farm and non-farm children was that nuts were introduced earlier in the diet of farm children, as compared to rural non-farm children (11 versus 15 months, *P *= 0.02; Table 2). Food diversity, measured as the number of food groups introduced by 6 months of age, did not differ between the farm and non-farm children (Table 2).

### Food-introduction practices in healthy and subsequently allergic children

One of 28 farm children (4%) and 10 of 37 non-farm children (27%) were diagnosed as allergic at 3 years of age. The distribution of allergy diagnoses are shown in Table 3.

**Table 3.** Allergy diagnoses at 3 years of age.

	Eczema	Asthma	Food allergy	ARC^a^
Farm children				
1	x			
Non-farm children				
1	x			
2	x			
3	x			
4	x			
5	x		x	
6	x	x		
7		x		
8		x		
9		x		x
10			x	

Diagnoses were made by trained pediatricians, using strict predefined protocols.

^a^Allergic rhinoconjunctivitis.

Medians and interquartile ranges for duration of breastfeeding, introduction of different food groups and food diversity at 6 months of age are presented in Table 4. The duration of exclusive breastfeeding was significantly longer in healthy than allergic children (4 versus 1 months, *P *= 0.01; Table 4), as was any breastfeeding (8 versus 6 months, *P *= 0.02; Table 4). Healthy children tended to have flour introduced earlier to their diet than allergic children (5 versus 6 months, *P *= 0.08; Table 4).

**Table 4.** Duration of breastfeeding and introduction of complementary foods in healthy and subsequently allergic children.

	Healthy (*n *= 44)		Allergic (*n *= 11)		
	Median	IQR	Median	IQR	
Variables	months		months		*P*
Exclusive breastfeeding	4	2–4	1	0–3	0.01
Any breastfeeding	8	5–12	6	2–7	0.02
Any food group	4	4–4	4	4–5	0.79
Potatoes	4	4–5	4	4–5	0.98
Vegetables	5	4–6	5	4–6	0.83
Legumes	7	6–8	6	5–7	0.16
Fruit	5	4–5	4	4–5	0.34
Berries	6	5–8	9	5–10	0.45
Peanuts	19	15–19	19	17–19	0.66
Nuts	12	11–18	19	11–19	0.13
Meat	6	5–6	5	5–6	0.37
Eggs	8	6–9	8	5–11	0.57
Fish	6	5–7	6	5–10	0.33
Flour	5	5–6	6	5–6	0.08
Milk	5	4–5	5	4–6	0.29
	Number of food items introduced				
Food diversity at age 6 months	8	6–9	8	6–9	0.72

The cumulative proportions of healthy and allergic children for whom formula or food have been introduced at different time-points is shown in Figure 2, including introduction of formula or any food (i.e. stopped being exclusively breastfed), any type of food, eggs, fish, and flour. Chi^2^ analyses were performed to compare the proportion of healthy and allergic children that had been introduced to formula or different food items at each time-point (Figure 1). Significant differences were found for introduction of formula or any food between healthy and allergic children at 1 month (18% versus 64%, respectively, *P *= 0.01), 2 months (27% versus 64%, respectively, *P *= 0.04) and 3 months (36% versus 82%, respectively, *P *= 0.02) in all subjects (Figure 1(a)). When the introduction of ‘any food’ was analyzed separately from ‘any food or formula’, the rate of food introduction was similar in healthy and allergic subjects (Figure 1(b)), suggesting that introduction of formula, and not food, accounted for the difference between subsequently healthy and allergic children. A higher proportion of the healthy children had received fish by 10 months of age (100% Healthy, 82% Allergic; *P *= 0.04; Figure 1(c)). Furthermore, a higher number of healthy subjects had received eggs at 10 months of age compared to allergic subjects (96% Healthy, 64% Allergic; *P *= 0.02; Figure 1(d)). We also found a tendency to a higher proportion of the healthy children having received flour both at 4 and 5 months of age, as compared to subjects who developed allergy (75% Healthy, 46% Allergic; *P *= 0.08; Figure 2(e)).

### Multivariable analysis of breastfeeding, food introduction, and allergy development

Logistic regression was performed to control the significant, or near significant, relationships for confounding, including exclusive breastfeeding and introduction of flour, fish, and eggs (independent variables) and being allergic at 3 years of age (dependent variable; Table 5). Exclusive breastfeeding and introduction of flour were entered as continuous variables, measured in months. Having introduced eggs and fish at/before 10 months of age or after was entered as a dichotomous variables, since significances were found only at this month. The model with fish was not robust for calculation of odds ratios; hence *P*-values from likelihood ratio tests are shown (Table 5). For flour, the original variable of month of introduction was converted to consumption before the age of 1.5 years (the last time-point for registration of food introduction). The conversion was made to display decreased risks of early introduction, rather than increased risks of late introduction. Due to the small number of allergic subjects, potential covariates were included in separate models.

**Table 5.** Logistic regression of allergy development, exclusive breastfeeding and introduction of flour, fish, and eggs.

Model	Potential covariates	Exclusive breastfeeding^b^	*P*	Flour^c^	*P*	Eggs ≤10 mo^d^	*P*	Fish ≤10 mo^d^ *P*^f^
1	Crude model	0.59 (0.39–0.89)	0.01	0.64 (0.32–1.24)	0.19	0.08^e^ (0.13–0.54)	0.01	0.01
2	Exclusive breastfeeding			0.42 (0.18–0.95)	0.04	0.08 (0.01–0.64)	0.02	0.02
3	Maternal heredity	0.59 (0.39–0.89)	0.013	0.58 (0.30–1.20)	0.15	0.08 (0.01–0.55)	0.011	0.004
4	Paternal smoking^a^	0.59 (0.39–0.89)	0.013	0.60 (0.30–1.19)	0.15	0.09 (0.01–0.59)	0.013	0.02
5	Cats/dogs in house at recruitment	0.51 (0.31–0.82)	0.01	0.64 (0.32–1.28)	0.21	0.09 (0.01–0.60)	0.013	0.003
6	Gestational week	0.61 (0.39–0.94)	0.02	0.64 (0.31–1.30)	0.22	0.09 (0.01–0.63)	0.02	0.01
7	Cesarean section	0.61 (0.40–0.92)	0.02	0.60 (0.30–1.19)	0.14	0.07 (0.01–0.48)	0.01	0.004
8	Birth weight	0.62 (0.41–0.94)	0.02	0.64 (0.31–1.30)	0.22	0.05 (0.01–0.40)	0.01	0.01
9	Male gender	0.60 (0.39–0.93)	0.02	0.42 (0.18–1.00)	0.051	0.04 (0.003–0.46)	0.01	0.004
10	Farm group	0.61 (0.40–0.95)	0.03	0.36 (0.13–1.01)	0.051	0.10 (0.01–0.80)	0.03	0.001

Analyses were carried out on significant or near significant relationships between allergy and breastfeeding or food-introduction practices. Owing to the low number of allergic subjects, each potential covariate was added in separately in the adjusted models, i.e. only one adjustment variable at a time.

*n *= 44 healthy and 11 subsequently allergic children.

^a^Smoking during the last month of pregnancy.

^b^Entered as a continous variable: months of exclusive breastfeeding.

^c^Entered as a continous variable: months of consumption within 1.5 year of age (the last time-point for registration of food introduction).

^d^Entered as a dicotomous variable: 1 = having eggs/fish introduced ≤10 months of age; 0 = having eggs/fish introduced >10 months.

^e^Hosmer and Lemeshow Test not applicable.

^f^Likelihood ratio test (χ^2^).

Longer exclusive breastfeeding was significantly associated with less allergy (crude OR: 0.59, 95% CI: 0.39–0.89, *P *= 0.01), and when adjusted for potential covariates (Table 5). The OR decreased marginally in the models including cats or dogs in the house (Table 5). Having introduced eggs at 10 months of age or earlier was also significantly associated with less allergy, compared to later introduction (crude OR: 0.08, 95% CI: 0.13–0.54, *P *= 0.01). The OR increased marginally when adjusted for paternal smoking, cats or dogs, gestational week, and group belonging, but decreased when adjusted for cesarean section, birth weight and male gender (Table 5). The *P*-value for the association between having introduced fish up through 10 months of age and less allergy increased from 0.01 to 0.02, when adjusted for exclusive breastfeeding (Table 5). For the rest of the potential covariates, the *P*-value remained the same or decreased to different extents with the lowest *P*-value observed when adjusted for farming/non-farming family (0.001, Table 5). Early introduction of flour was significantly negatively associated with being allergic at the age of 3 years only when adjusted for exclusive breastfeeding (OR: 0.42, 95% CI: 0.18–0.95, *P *= 0.04), and borderline significant when adjusted for gender and farming/non-farming family (Table 5).

### Sensitivity analysis

The decision to exclude children with transient allergy at 1.5 years of age leaves a lower number of children in the allergic group and reduces the power compared to if cumulative allergy at 3 years of age would have been the outcome. Hence, sensitivity analyses of relevant variables from the original analyses were carried out in retrospect to evaluate whether the selection decision changes the conclusion. Early exclusive breastfeeding remained significantly inversely associated with less allergy, also when adjusted for farming status, although significant only up to 1 month of age. The inverse association with allergy remained significant in the crude analyses of introduction of eggs and fish, but when adjusted for farming status the association between egg introduction and allergy turned into a tendency. Introduction of flour was significantly associated with allergy only when adjusted for exclusive breastfeeding in the original analysis. In the sensitivity analysis, the significance was lost in the model adjusted for breastfeeding, although the tendency remained in the crude model.

## Discussion

As previously reported in the FARMFLORA birth cohort, a lower prevalence of allergy at 3 years of age was observed in the farm children compared to the rural resident non-farm children [3]. Only one farm child was allergic at 3 years of age, as compared with ten in the non-farm group. The protective effect of growing up on a small, family-owned dairy farm is in line with previous studies [2,4,36]. Recent randomized trials point to postponement of introduction of solid foods as a strong risk factor for development of food allergy [15,16] and several observational studies have pointed to delayed introduction of fish as predictive of high risk of becoming allergic [22–29]. To the best of our knowledge, practices of introducing complementary foods have not been investigated previously in children of farming families. The aim of the present study was to investigate if differences in food-introduction practices between farm and non-farm children may explain part of the protective effect observed in children growing up in a farming environment.

We found that farming families practiced slightly longer breastfeeding than did control families. The differences in the timing of introduction of different food groups between farming and control families were quite small, and the food diversity at 6 months of age was very similar. However, nuts were introduced significantly earlier to the diet of farm children, and peanuts tended to be introduced earlier among the farmers. Delayed introduction of nuts [37], and peanuts [15,16], have previously been linked to an increased risk of developing allergy. However, the 0.01 < 0.05 *P*-value may be subjected to type I errors due to multiple comparisons. Moreover, nuts were introduced only insignificantly later in subsequently allergic children compared to healthy children in this study and peanuts were introduced at the same age in both healthy and subsequently allergic children. Hence, earlier introduction of nuts or any other food group is unlikely to explain the lower rate of allergy development at 3 years of age among the farm children in our study.

Having introduced fish at 10 months of age or earlier was significantly associated with less allergy in our study. As mentioned above, early introduction of fish has been associated in several studies with a reduced risk of allergy development [22–29]. Our most robust finding was, however, a lower rate of allergy in children that had consumed eggs at 10 months of age or earlier, compared to children who received eggs later than 10 months of age. This finding is in line with Nwaru and coworkers, who showed that introduction of eggs after 11 months of age was associated with an increased risk of asthma, allergic rhinitis, and atopic sensitization at 5 years of age compared to introduction up through 11 months of age [29]. Moreover, Filipiak and coworkers observed a higher risk of eczema in the first 4 years in children who had eggs introduced after 1 year of age [37]. In a large, randomized, controlled intervention of three-month-old infants, early introduction of eggs reduced the risk of egg allergy [16].

In the logistic regression model, a significantly lower rate of allergy was found among those children who got flour early, when adjusted for exclusive breastfeeding. According to the χ^2^-tests, an insignificantly higher rate of healthy children had flour introduced up through the ages of 4 and 5 months compared with subsequently allergic children. Although wheat allergy and celiac disease are two different types of immune-mediated hypersensitivity reactions, a meta-analysis has shown a 25% increased risk of developing celiac disease if gluten is introduced after 6 months of age, as compared with introduction between 4 and 6 months of age [38]. On the other hand, the timing of introduction of gluten was not found to affect the development of celiac disease in two large randomized controlled trials of high-risk infants [39,40].

Despite our findings that indicated adverse effects of introducing certain foods late, we observed a shorter duration of both exclusive and any breastfeeding in children who became allergic. A significantly lower rate of the allergic children in this study was breastfed exclusively up to the age of 3 months compared with children who remained healthy. According to a recently published review article by Heinrich, results regarding breastfeeding in the context of allergy prevention are mixed and do not provide a clear picture [41]. He concludes that current guidelines of exclusive breastfeeding for 4–6 months for the prevention of allergies are not supported [41].

While breastfeeding up to 3 months of age appeared protective in our study, it did not appear as though prolonged exclusive breastfeeding beyond 3 months was advantageous. Prolonged exclusive breastfeeding may deprive the infant of foreign proteins to which tolerance should be developed. Thus, it is important to distinguish between potential positive effects of receiving breast milk and potentially detrimental effects of being shielded from harmless exogenous proteins to which one should develop immune tolerance. Being fed a protein is the most effective way of becoming tolerant. Gideon Lack has proposed the dual exposure hypothesis: exclusion of foreign proteins from the infant’s diet means that one is primarily exposed via other routes, i.e. the cutaneous or inhalant route, which may result in sensitization and allergy development, rather than tolerance [42,43]. Dietary proteins normally induce antigen-specific active immune tolerance, termed oral tolerance, as observed in experimental animals [44,45] as well as in humans [46]. Animal experiments have shown that feeding a sufficient amount of the foreign protein is required to achieve tolerance, while feeding smaller doses may instead result in increased sensitization [17,18].

Owing to previous recommendations of postponing the introduction of allergenic foods in high-risk infants [12], reverse causation has become a major problem in studies of timing of food introduction and allergy development. Hence, foods may be introduced later to children who are already allergic [27,47,48] or have a family history of allergy [47,49]. However, we found no indications of reverse causation in our study based on a questionnaire sent to the parents in retrospect and, thus, we did not perform additional analyses on this matter.

The small sample size of this cohort limits the statistical power. This may partly explain the lack of strong significant results in our study, considering the coherence with previously published studies. Still, the results must be interpreted with caution, not least due to multiple comparisons. However, the size of the cohort has permitted thorough clinical doctors’ diagnoses of allergy, which is a strength, although it was not possible to use different atopic manifestations as outcomes because of the low number of allergic children. Larger studies are needed that include very early and consistent introduction of a broad variety of well-defined food groups as well as well-defined clinical diagnoses of allergy.

In conclusion, differences in food-introduction practices between farm and non-farm families do not seem to explain the lower rate of subsequently allergy development in the farm children in this study. Although unrelated to farming status, our results point in the same direction as a growing number of studies indicating that postponed introduction of complementary foods is not beneficial, but might instead increase the risk of allergy development. Further, a beneficial effect of breastfeeding in the first months of life does not contradict a potentially negative effect of prolonged exclusive breastfeeding, resulting in a delayed introduction of foods However, owing to the limited sample size and our explorative approach with multiple comparisons, these results are only indicative.
